# Closed suction drainage following routine primary total joint arthroplasty is associated with a higher transfusion rate and longer postoperative length of stay: a retrospective cohort study

**DOI:** 10.1186/s13018-019-1211-0

**Published:** 2019-05-29

**Authors:** Hong Xu, Jinwei Xie, Yiting Lei, Qiang Huang, Zeyu Huang, Fuxing Pei

**Affiliations:** Department of Orthopedic Surgery, West China Hospital, Sichuan University, Chengdu, 610041 China

**Keywords:** Knee, Hip, Arthroplasty, Transfusion, Length of stay, Drain

## Abstract

**Background:**

In an enhanced recovery after surgery program, not placing a closed suction drain following routine primary total joint arthroplasty (TJA) is becoming more acceptable. However, the influence of drain use on transfusion rate and postoperative length of stay (PLOS) in TJA remains controversial. Therefore, we aimed to compare drain use with no drain in routine primary TJA to determine the differences in transfusion rate and PLOS.

**Methods:**

We analyzed the data from 12,992 patients undergoing primary unilateral TJA: 6325 total knee arthroplasties (TKA) and 6667 total hip arthroplasties (THA). Patients were divided into two groups according to whether they received a drain postoperatively following TKA and THA. We extracted information for transfusion and PLOS from patients’ electronic health records and analyzed the data by logistic and linear regression analyses.

**Results:**

The transfusion rate and PLOS were 15.07% and 7.75 ± 3.61 days, respectively, in the drain group and 6.72% and 6.54 ± 3.32 days, respectively, in the no-drain group following TKA. The transfusion rate and PLOS were 20.53% and 7.00 ± 3.35 days, respectively, in the drain group and 13.57% and 6.07 ± 3.06 days, respectively, in the no-drain group following THA. After adjusting for the following variables: age, gender, body mass index, orthopedic diagnoses, hypertension, type 2 diabetes, coronary heart disease, chronic obstructive pulmonary disease, preoperative hemoglobin, albumin, analgesic use, anesthesia, American Society of Anesthesiologists class, tranexamic acid use, intraoperative bleeding, operative time, and tourniquet use (for TKA), drain use correlated significantly with a higher transfusion rate (risk ratio = 2.812, 95% confidence interval (CI) 2.224–3.554, *P* < 0.001 for TKA and risk ratio = 1.872, 95% CI 1.588–2.207, *P* < 0.001 for THA) and a longer PLOS (partial regression coefficient (*B*) = 1.099, 95% CI 0.879–1.318, *P* < 0.001, standard regression coefficient (*B*′) = 0.139 for TKA; *B* = 0.973, 95% CI 0.695–1.051, *P* < 0.001, and *B*′ = 0.115 for THA). Two groups showed no significant difference in wound complications.

**Conclusions:**

Our findings indicated that drain use was associated with a higher transfusion rate and a longer PLOS in patients undergoing routine primary TJA. The routine use of postoperative drainage is not recommended in primary unilateral TJA.

## Introduction

The use of closed suction drainage (CSD) in surgery has been a standard practice since the time of Hippocrates [1]. Many surgeons follow the practice based on their early training, rather than on the basis of scientific evidence [2]. Proponents believe that postoperative CSD reduces ecchymosis [3] and avoids hematoma formation, which impair wound healing by increasing wound tension and decreasing surrounding tissue blood perfusion [4]. In addition, a hematoma also may lead to superficial and deep-seated infection secondary to bacterial colonization [5]. However, the exclusion of CSD in total joint arthroplasty (TJA) is becoming increasingly accepted with enhanced recovery after surgery (ERAS) programs. Opponents of CSD propose that CSD in TJA is associated with greater blood loss because the drain prevents the tamponade effect [6] and because of the higher risk of infection secondary to retrograde bacterial migration [7]. Moreover, some studies [8, 9] also reported that the use of CSD impairs early postoperative rehabilitation and complicates postoperative nursing care.

Although some previous studies have evaluated the effect of CSD on transfusion rate and postoperative length of stay (PLOS) in total knee arthroplasty (TKA) and total hip arthroplasty (THA), CSD remains controversial because most studies had insufficient sample sizes [10]. Therefore, we based this study on a large dataset to investigate (1) whether CSD increases the postoperative transfusion rate, and (2) whether CSD is associated with a longer PLOS after routine primary TKA and THA.

## Materials and methods

### Data source

This study was a secondary analysis of a large database generated from a prospective multicenter study evaluating the efficacy and safety of perioperative management of TKA and THA. This study protocol was registered in the Chinese Clinical Trial Registry. The surgeons decided whether to use CSD at the end of surgery. Transfusion criteria were (1) hemoglobin level < 70 g/L and (2) hemoglobin level 70–100 g/L with symptomatic anemia appearing as lightheadedness, presyncope, fatigue, palpitation, or shortness of breath, excluding other causes. We defined PLOS data as the number of days between the date of surgery and the date of discharge. The database used in this study included patient-level hospital discharge data, which were provided by 26 university teaching hospitals in China (10 national and 16 regional hospitals) sponsored by the Chinese Health Ministry. The completeness and validity of the data were confirmed by careful comparison with data from hospital information systems. Patient consent was deemed unnecessary by each hospital’s institutional review board.

We recorded the following patient information: demographic characteristics, preoperative comorbidities and drug use, laboratory values, operative variables, and wound complications. Demographic characteristics comprised age, gender, body mass index (BMI), and orthopedic diagnoses. Preoperative comorbidities and drug use included hypertension, type 2 diabetes, chronic obstructive pulmonary disease (COPD), coronary heart disease, and preoperative analgesic use. Preoperative laboratory values comprised preoperative hemoglobin and albumin. We recorded the following operative variables: method of anesthesia (general or regional anesthesia), American Society of Anesthesiologists (ASA) class evaluated by an anesthetist preoperatively, the use of tranexamic acid (TXA) and drainage, operative time, intraoperative bleeding, and tourniquet use in TKA. Wound complications included non-infectious complications (fat liquefies and wound effusion, wound necrosis and hematoma) and infectious complications [superficial wound infection and periprosthetic joint infection (PJI) within a month postoperatively].

### Patients’ demographics

All patients, namely, 6325 patients undergoing routine primary TKA and 6667 undergoing routine primary THA, were identified by the presence of the International Classification of Diseases, Tenth Revision, Clinical Modification procedure codes. Based on discharge records, we excluded patients who had undergone previous arthroplasty or bilateral TKA or THA, patients with metastatic and/or bone cancer, and those experiencing a joint dislocation. We also excluded patients younger than age 18 years or who received an autologous blood transfusion rather than an allogeneic blood product. Patients undergoing TKA were divided into two groups based on the presence of degenerative arthritis (osteoarthritis) and inflammatory arthritis of the knee into a rheumatoid arthritis group and an ankylosing spondylitis group. We divided patients undergoing routine primary THA into three groups: a degenerative arthritis group (osteoarthritis, osteonecrosis of the femoral head, and developmental dysplasia of the hip), an inflammatory arthritis group (rheumatoid arthritis and ankylosing spondylitis of the hip joint), and a group with femoral neck fracture.

### Statistical analyses

Variables were summarized as mean ± standard deviation for continuous variables and frequency (proportion) for categorical variables. We built a crude model of transfusion rate and PLOS regarding the use of CSD, using univariate analyses. We used logistic and linear regression analyses to adjust for other variables and explore the relationships between the variables. Statistical significance was set as *P* < 0.05. All data analyses were performed using SPSS version 21 software (IBM, Armonk, NY, USA).

## Results

### Patients’ demographics

The demographics for patients who underwent routine primary TKA are shown in Table 1. The TKA group included 4540 patients in the drain group and 1785 patients in the no-drain group. The total transfusion rate was 12.71%: 15.07% in the drain group and 6.72% in the no-drain group. The average PLOS was 7.41 ± 3.57 days after TKA: 7.75 ± 3.61 days in the drain group and 6.54 ± 3.32 days in the no-drain group (Table 2).

**Table 1:** The demographic characteristics of the patients who underwent routine primary TKA

Baseline characteristic	Drain use (*n* = 4540)	No drain (*n* = 1785)	All (*n* = 6325)
Demographic characteristics			
Age (*X* ± *S*)	66.56 ± 8.79	66.68 ± 8.64	66.60 ± 8.75
Female, *N* (%)	3613 (79.58%)	1358 (76.08%)	4971 (78.59%)
BMI (kg/m^2^)	25.84 ± 5.48	25.04 ± 3.56	25.62 ± 5.02
Diagnosis, *N* (%)			
OA	4241 (93.41%)	1683 (94.29%)	5924 (93.66%)
Inflammatory arthritis	299 (6.59%)	102 (5.71%)	401 (63.40%)
Comorbidities, *N* (%)			
Hypertension	1351 (29.76%)	356 (19.94%)	1707 (26.99%)
Type 2 diabetes	360 (7.93%)	73 (4.09%)	433 (6.85%)
CHD	182 (4.01%)	35 (1.96%)	217 (3.43%)
COPD	24 (0.53%)	10 (0.56%)	34 (0.54%)

*BMI* body mass index, *OA* osteoarthritis, *CHD* coronary heart disease, *COPD* chronic obstructive pulmonary disease

**Table 2:** The transfusion rate and postoperative length of stay in routine primary TKA

	Drain use (*n* = 4540)	No drain (*n* = 1785)	All (*n* = 6325)
Transfusion, *N* (%)	684 (15.07%)	120 (6.72%)	804 (12.71%)
PLOS (days)	7.75 ± 3.61	6.54 ± 3.32	7.41 ± 3.57

*PLOS* postoperative length of stay

The demographics for patients who underwent routine primary THA are shown in Table 3. The THA group included 4943 patients in the drain group and 1724 patients in the no-drain group. The total transfusion rate was 18.74%: 20.53% in the drain group and 13.57% in the no-drain group. The average PLOS was 6.76 ± 3.30 days: 7.00 ± 3.35 days in the drain group and 6.07 ± 3.06 days in the no-drain group (Table 4).

**Table 3:** The demographic characteristics of the patients who underwent routine primary THA

Baseline characteristic	Drain use (*n* = 4943)	No drain (*n* = 1724)	All (*n* = 6667)
Demographic characteristics			
Age (*X* ± *S*)	58.31 ± 13.62	58.61 ± 14.34	58.39 ± 13.81
Female, *N* (%)	2654 (53.69%)	988 (57.31%)	3642 (54.63%)
BMI (kg/m^2^)	23.69 ± 3.37	23.79 ± 4.72	23.71 ± 4.41
Diagnosis, *N* (%)			
OA	4033 (81.59%)	1392 (80.74%)	5425 (81.37%)
Inflammatory arthritis	199 (4.03%)	67 (3.89%)	266 (3.99%)
Fracture of femoral neck	711 (14.38%)	265 (15.37%)	976 (14.64%)
Comorbidities, *N* (%)			
Hypertension	624 (12.62%)	262 (15.20%)	886 (13.29%)
Type 2 diabetes	171 (3.46%)	56 (3.25%)	227 (3.40%)
CHD	55 (1.13%)	32 (1.86%)	87 (1.30%)
COPD	19 (0.38%)	7 (0.41%)	26 (0.39%)

*BMI* body mass index, *OA* osteoarthritis, *CHD* coronary heart disease, *COPD* chronic obstructive pulmonary disease

**Table 4:** The transfusion rate and postoperative length of stay in routine primary THA

	Drain use (*n* = 4943)	No drain (*n* = 1724)	All (*n* = 6667)
Transfusion, *N* (%)	1015 (20.53%)	234 (13.57%)	1249 (18.74%)
PLOS (days)	7.00 ± 3.35	6.07 ± 3.06	6.76 ± 3.30

*PLOS* postoperative length of stay

### The effect of drain use on transfusion rate and PLOS after TKA

As shown in Table 5, univariate analyses (crude model) showed that CSD was highly associated with the transfusion rate (relative risk (RR) = 2.461, 95% confidence interval (CI) 2.010–3.013, *P* < 0.001) and PLOS (partial regression coefficient (*B*) = 1.214, 95% CI 1.016–1.412, *P* < 0.001; standard regression coefficient (*B*′) = 0.153) after TKA. After controlling for age, gender, BMI, and orthopedic diagnoses (model 1), logistic and linear regression analyses showed that CSD remained correlated with a higher transfusion rate (RR = 2.561, 95% CI 2.090–3.139, *P* < 0.001) and longer PLOS (*B* = 1.223, 95% CI 1.025–1.422, *P* < 0.001; *B*′ = 0.154).

**Table 5:** The result of logistic and linear regression analyses in TKA

	Transfusion rate		PLOS		
	OR (95% CI)	*P*	*B* (95% CI)	*P*	*B*′
Crude model	2.461 (2.010–3.013)	0.000	1.214 (1.016–1.412)	0.000	0.153
Model 1	2.561 (2.090–3.139)	0.000	1.223 (1.025–1.422)	0.000	0.154
Model 2	2.529 (2.052–3.116)	0.000	1.492 (1.290–1.694)	0.000	0.188
Model 3	2.812 (2.224–3.554)	0.000	1.099 (0.879–1.318)	0.000	0.139

*PLOS* postoperative length of stay

Model 1: Controlled for age, gender, body mass index, MI, and diagnoses

Model 2: Controlled for model 1 + hypertension, type 2 diabetes, chronic obstructive pulmonary disease, coronary heart disease, preoperative analgesic use, hemoglobin, and albumin

Model 3: Controlled for model 2 + method of anesthesia, American Society of Anesthesiologists class, operative time, intraoperative bleeding, tranexamic acid, and tourniquet use

After additional adjustment for hypertension, type 2 diabetes, COPD, coronary heart disease, preoperative analgesic use, and preoperative hemoglobin and albumin (model 2), CSD remained associated with a higher transfusion rate (RR = 2.529, 95% CI 2.052–3.116, *P* < 0.001) and a longer PLOS (*B* = 1.492, 95% CI 1.290–1.694, *P* < 0.001; *B*′ = 0.188). After further controlling for all covariates (covariates in model 2 plus the method of anesthesia, ASA class, operative time, intraoperative bleeding, and TXA and tourniquet use), drain use remained correlated with a higher transfusion rate (odds ratio (OR) = 2.812, 95% CI 2.224–3.554, *P* < 0.001) and a longer PLOS (*B* = 1.099, 95% CI 0.879–1.318, *P* < 0.001; *B*′ = 0.139), as in model 3.

### The effect of drain use on transfusion rate and PLOS after THA

As shown in Table 6, univariate analyses (crude model) revealed that CSD was correlated with higher transfusion rates (RR = 1.645, 95% CI 1.410–1.920, *P* < 0.001) and longer PLOS (*B* = 0.927, 95% CI 0.734–1.111, *P* < 0.001; *B*′ = 0.123) after THA. Logistic and linear regression analyses showed that CSD was correlated with a higher transfusion rate (RR = 1.657, 95% CI 1.420–1.935, *P* < 0.001) and a longer PLOS (*B* = 0.935, 95% CI 0.753–1.117, *P* < 0.001; *B*′ = 0.123) after controlling for age, gender, BMI, and orthopedic diagnoses (model 1). After further adjustment for hypertension, type 2 diabetes, COPD, coronary heart disease, preoperative analgesic use, and hemoglobin and albumin level (model 2), CSD remained associated with a higher transfusion rate (RR = 1.690, 95% CI 1.445–1.978, *P* < 0.001) and a longer PLOS (*B* = 0.931, 95% CI 0.750–1.112, *P* < 0.001; *B*′ = 0.122). After controlling for all covariates, which included the covariates in model 2 plus the method of anesthesia, ASA class, operative time, intraoperative bleeding, and TXA use, CSD remained correlated with a higher transfusion rate (OR = 1.872, 95% CI 1.588–2.207, *P* < 0.001) and longer PLOS (*B* = 0.973, 95% CI 0.695–1.051, *P* < 0.001; *B*′ = 0.115), as in model 3.

**Table 6:** The result of logistic and linear regression analyses in THA

	Transfusion		PLOS		
	OR (95% CI)	*P*	*B* (95% CI)	*P*	*B*′
Crude model	1.645 (1.410–1.920)	0.000	0.927 (0.734–1.111)	0.000	0.123
Model 1	1.657 (1.420–1.935)	0.000	0.935 (0.753–1.117)	0.000	0.123
Model 2	1.690 (1.445–1.978)	0.000	0.931 (0.750–1.112)	0.000	0.122
Model 3	1.872 (1.588–2.207)	0.000	0.973 (0.695–1.051)	0.000	0.115

*PLOS* postoperative length of stay

Model 1: controlled for age, gender, body mass index, MI, and diagnoses

Model 2: controlled for model 1 + hypertension, type 2 diabetes, chronic obstructive pulmonary disease, coronary heart disease, preoperative analgesic use, hemoglobin, and albumin

Model 3: controlled for model 2 + method of anesthesia, American Society of Anesthesiologists class, operative time, intraoperative bleeding, and tranexamic acid

### The wound complication rates between the two groups in TKA and THA

As shown in Tables 7 and 8, there are no differences in the non-infectious and infectious complication rates between the drain and no-drain group after routine primary TKA and THA (*P* > 0.05).

**Table 7:** The wound complication rates between the two groups in routine primary TKA

	Drain use (*n* = 4540)	No drain (*n* = 1785)	*P* value
Non-infectious complications, *N* (%)			
Fat liquefies and wound effusion	183 (4.03%)	74 (4.15%)	0.835%
Wound necrosis	69 (1.52%)	26 (1.46%)	0.852%
Hematoma	112 (2.47%)	48 (2.69%)	0.613%
Infectious complications, *N* (%)			
Superficial wound infection	59 (1.23%)	21 (1.18%)	0.693%
PJI within a month postoperatively	4 (0.09%)	1 (0.06%)	1.000%

*PJI* periprosthetic joint infection

**Table 8:** The wound complication rates between the two groups in routine primary THA

	Drain use (*n* = 4943)	No drain (*n* = 1724)	*P* value
Non-infectious complications, *N* (%)			
Fat liquefies and wound effusion	186 (3.76%)	63 (3.65%)	0.838%
Wound necrosis	47 (0.95%)	19 (1.10%)	0.585%
Hematoma	103 (2.08%)	37 (2.15%)	0.876%
Infectious complications, *N* (%)			
Superficial wound infection	52 (1.03%)	19 (1.10%)	0.861%
PJI within a month postoperatively	4 (0.08%)	2 (0.12%)	1.000%

*PJI* periprosthetic joint infection

## Discussion

This study evaluating data for 12,992 patients was performed to provide conclusive results regarding the effect of CSD in patients undergoing primary unilateral TJA on transfusion rate and PLOS. The most important finding was that CSD was associated with a higher transfusion rate and a longer PLOS in patients undergoing routine primary TKA and THA.

TJA is a successful and common surgical treatment for patients with end-stage knee or hip diseases because it can significantly relieve pain and improve patients’ function and quality of life [11]. Although efforts have been made to reduce perioperative blood loss, significant early postoperative total blood loss of up to 1000–1500 mL including intraoperative bleeding and hidden blood loss remains a challenge [12]. A previous study [13] based on a nationwide inpatient sample database reported that TKA and THA were among the top 10 most-frequent procedures requiring transfusion in the USA. Furthermore, blood transfusion was related to numerous serious complications such as viral infection, anaphylactic and hemolytic reactions, longer PLOS, deep vein thromboembolism, high hospitalization costs, and increased in-hospital mortality [14–16]. Allogeneic blood reserves are often insufficient worldwide; consequently, reducing the transfusion rate is essential for patients undergoing TKA or THA.

Previous studies [2, 9, 17, 18] have demonstrated that CSD was not associated with a higher transfusion rate in primary unilateral TJA; however, this finding may be related to the small sample sizes and insufficient data in these studies. A systematic review and meta-analysis [19] evaluating 19 randomized controlled trials reported that CSD significantly increased the risk of transfusion in primary TKA (RR = 1.38, 95% CI 1.04–1.83); we found similar results (OR = 2.812, 95% CI 2.224–3.554 in TKA; RR = 1.872, 95% CI 1.588–2.207 in THA). Furthermore, Bjerke-Kroll et al. [20] reported that CSD increased the number of allogeneic blood transfusions both in unilateral primary TKA and in THA, possibly because of the higher blood loss in the drain group. Several previous studies [2, 8, 20–22] reported that CSD was associated with increased total blood loss in TKA and THA. Studies [23, 24] have also revealed that hidden blood loss accounts for approximately 60% of total blood loss in TJA and the majority of the blood loss happens early postoperatively. Hidden blood loss occurs primarily because of large blood accumulation in the joint space and muscle compartments, and when a drain is placed between the joint space and muscle compartment postoperatively, bleeding continues until the drain is removed because the drain prevents the tamponade effect, which is an important step in filling the dead space in an operated wound [1, 25].

LOS is considered both a significant outcome measure when evaluating ERAS programs [13] and a non-substitutable marker of excellence [26]. Reducing LOS, which means improved hospital efficiency and productivity, is an important aim in many counties. Shorter LOS has obvious benefits for both hospitals and patients, namely, lower use of medical resources, significant cost savings, and excellent patient satisfaction [27, 28]. Shortening PLOS has become a vital part of shortening LOS with the development of ERAS programs. Despite some previous studies [2, 9, 17, 19] reporting that CSD was not associated with a longer PLOS, the routine use of CSD was not recommended by these studies because this empirical practice has no obvious advantages for patients undergoing primary unilateral TKA or THA. Furthermore, Sharma et al. [1] and Bjerke-Kroll et al. [20] reported that LOS was longer in their drain groups compared with the no-drain groups after TKA and THA, respectively. Our results also showed that CSD was associated with a longer PLOS after both TKA and THA. CSD interferes with early mobilization and complicates postoperative nursing care, which may contribute to the longer PLOS after TKA or THA [8, 21].

Wound complication, especially for infectious complications, would bring catastrophic consequences. Besides, non-infectious complications and superficial wound infection also may develop into PJI if they are not handled properly, which is one of the most devastating complications after TJA [29]. However, our study showed there are no differences in wound complications between the drain and no-drain groups after routine primary TKA and THA. Zhang et al. [30] conducted a systematic review and meta-analysis which enrolled 19 randomized controlled trials that showed drain use was not correlated with superficial wound infection and PJI in primary TKA, which was in accordance with our study. However, Willemen et al. [31] carried out a prospective randomized controlled trial which enrolled 41 patients who undergone TKA showing that bacteria cultures of all drain tips with cutoff at postoperative 24 h were negative, while 5 of 21 drain tips indwelling for 48 h after operation developed positive culture results (all bacteria were *Staphylococci*), which indicated that CSD may be a source of retrograde infection and the risk increased with the extension of indwelling time. Hence, future studies are needed to estimate the effect of CSD on wound complication, especially for infectious complications.

CSD use in TJA should balance the advantages and disadvantages, which depend mainly on the patient’s clinical condition. With the development of ERAS programs worldwide, CSD has been reconsidered by orthopedic surgeons, and excluding CSD in routine primary TKA and THA is becoming more popular [2]. Although the purchase cost of a drainage tube is relatively low, longer PLOS and higher transfusion rates caused by CSD may increase total hospitalization costs for patients undergoing TJA [20]. In addition, CSD may delay patients’ postoperative recovery because CSD interferes with early mobilization and complicates nursing care, despite the need for fewer postoperative dressing changes [9, 32]. In addition, with the associated longer incisions, longer surgery times, more bleeding, and potential for hematoma formation, revision TKA and THA are considered more complex procedures compared with routine primary TJA [33]. Abolghasemian et al. [34] reported that CSD in revision knee arthroplasty was associated with higher blood loss and a higher transfusion rate, and no differences were found regarding postoperative wound complications and knee scores compared with the no-drain group. Fichman et al. [35] also reported that CSD in revision TJA was correlated with a higher transfusion rate and a longer PLOS. Even so, future high-quality studies with longer follow-up are still needed to make a definitive conclusion regarding CSD in revision joint arthroplasty.

This study has several limitations. First, whether the drain was clamped and the clamping time were not recorded in detail in the database; thus, we were unable to determine the effect of drain clamping on transfusion rate and PLOS. Second, the drain removal criteria were not recorded, which may have affected the blood loss volume. Third, some important outcomes such as patients’ postoperative range of motion in the operated joint and the need for dressing changes were not recorded or evaluated. Fourth, the details of TXA use, other than yes or no, were not recorded in the database, and the starting time for TXA differed for each hospital, which may have affected the blood loss volume and transfusion rate. Despite these limitations, to the best of our best knowledge, our study included the largest number of patients to date and provided conclusive results regarding the use of CSD in TJA and its effect on transfusion rate and PLOS. The results of this study could become an important component of orthopedic surgeons’ decision-making regarding CSD in routine primary TKA and THA.

## Conclusion

In summary, the current study found that CSD was associated with a significantly higher transfusion rate and a longer PLOS in patients undergoing primary unilateral TKA and THA. We do not recommend the routine use of CSD in primary unilateral TJA.

## Data Availability

Please contact the author for data requests.

## Abbreviations

ASA: American Society of Anesthesiologists
*B*: Partial regression coefficient
*B*′: Standard regression coefficient
BMI: Body mass index
CI: Confidence interval
COPD: Chronic obstructive pulmonary disease
CSD: Closed suction drainage
ERAS: Enhanced recovery after surgery
OR: Odds ratio
PJI: Periprosthetic joint infection
PLOS: Postoperative length of stay
RR: Relative risk
THA: Total hip arthroplasty
TJA: Total joint arthroplasty
TKA: Total knee arthroplasty
